# Editorial commentary on the special issue of glia and neurological diseases

**DOI:** 10.7555/JBR.36.20220800

**Published:** 2022-09-28

**Authors:** Fengfei Ding

**Affiliations:** Department of Pharmacology, School of Basic Medical Sciences, State Key Laboratory of Medical Neurobiology and Ministry of Education Frontiers Center for Brain Science, Institutes of Brain Science, Shanghai Medical College, Fudan University, Shanghai 200032, China

The glia are the cells in the central nervous system (CNS) that originally were considered to only provide support, protection, and nutrition for neurons. The glia outnumber the neurons in the brain and spinal cord, the ratio of these two types of cells varies across species and tissues. The word "glia" comes from "glue", describing the cells that surround neurons and gathering them together like glue. However, in the past decades, studies gradually revealed the key roles of the glia and their heterogeneity in term of categorie and function.

In CNS, astrocytes make up to 20% to 40% population of the glia, while oligodendrocytes 40% to 70%, and microglia 10% to 20%. Astrocytes are highly heterogeneous in morphology and function, dominantly participating in the biological processes such as the homeostatic regulation of neurotransmitters, water, ions and pH; the regulation of energy balance, food intake and metabolism; sleep homeostasis, development and synaptic plasticity, as well as local blood flow regulation, perivascular waste clearance, *etc*. Oligodendrocytes, the mature categories of oligodendrocyte lineage cells in CNS, form the membranous sheath covering axons and facilitate the neuronal electric signal transduction. The pathogenic mechanisms, regeneration and repair of the demyelination lesions have been extensively studied in neurological diseases. Microglia, the primary innate immune effector cells of the CNS, are distinctive from the other types of glia in their origins and functions.

The exploration of the mystery of the glia has been aided by the technical development targeting in this field, particularly the techniques characterizing the glial functions in live animals. Contradicting conclusions were reported between *in vitro* or *ex vivo* and *in vivo* studies. The discrepancies hinted that the interstitial microenvironment and the interactions with neurons are vital for the pathophysiological properties of the glia. *In vivo* multi-photon imaging can reveal the morphological and functional features of *in situ* glial cells with cellular resolution. Positron emission tomography (PET) can be used to visualize the metabolic processes of the glia noninvasively with cell-type oriented sensors; finally, the development of molecular manipulation techniques, such as, opto- and chemo-genetics, genetically encoded fluorescent sensors for calcium signaling, neurotransmitters, substantially opened a new era of understanding with regard to the molecular mechanisms in glial cells.

In this special issue, we have selected eight different articles that are presenting recent advances in the study of the glia and their effect on health and disease, particularly focusing on astrocytes and oligodendrocytes. Obara-Michlewska reviewed the contribution of astrocytes to obesity-associated metabolic disturbances, including the central regulation of appetite and energy metabolism^[1]^. Zheng *et al* reviewed the latest progress in spinal cord tissue repair by transplantation of human astrocytes of various origins, including fetal brain, spinal cord and pluripotent stem cells^[2]^. Jia *et al* summarized the frontier progress in glial cell-type oriented tracers for PET imaging^[3]^. Gong *et al* summarized the recent studies about the dysfunction of oligodendrocytes in amyotrophic lateral sclerosis^[4]^. Li *et al* reviewed the immunologic role of oligodendrocyte lineage cells in demyelination diseases^[5]^. Yan *et al *summarized mechanisms regulating cerebral hypoperfusion in cerebral autosomal dominant arteriopathy with subcortical infarcts and leukoencephalopathy (CADASIL)^[6]^. Sun *et al* presented a visualization pipeline for *in vivo* two-photon volumetric astrocytic calcium imaging. Different from conventional two-dimensional imaging, they captured the three-dimensional cortical astrocytic Ca^2+^ time-lapse images through chronic craniectomy in brain state transitions from resting awake to isoflurane anesthetized states^[7]^. Wang *et al* reported a clinical study of six patients of CADASIL and twelve healthy controls, they found that the severity of hypoperfusion correlates with the white matter hyperintensity volume. It highlighted the mutual links between the elements of the glio-vascular unit (G-unit)^[8]^. We hope the readers find this special issue interesting and intriguing.
